# What is the Prevalence and Success of Remediation of Emergency Medicine Residents?

**DOI:** 10.5811/westjem.2015.9.27357

**Published:** 2015-10-22

**Authors:** Mark Silverberg, Moshe Weizberg, Tiffany Murano, Jessica L. Smith, John C. Burkhardt, Sally A. Santen

**Affiliations:** * SUNY Downstate/Kings County Hospital, Department of Emergency Medicine, New York, New York; † Staten Island University Hospital, Department of Emergency Medicine, Staten Island, New York; ‡ Rutgers New Jersey Medical School, Department of Emergency Medicine, Newark, New Jersey; § Alpert Medical School of Brown University, Department of Emergency Medicine, Providence, Rhode Island; ¶ University of Michigan Medical School, Department of Emergency Medicine, Ann Arbor, Michigan

## Abstract

**Introduction:**

The primary objective of this study was to determine the prevalence of remediation, competency domains for remediation, the length, and success rates of remediation in emergency medicine (EM).

**Methods:**

We developed the survey in Surveymonkey™ with attention to content and response process validity. EM program directors responded how many residents had been placed on remediation in the last three years. Details regarding the remediation were collected including indication, length and success. We reported descriptive data and estimated a multinomial logistic regression model.

**Results:**

We obtained 126/158 responses (79.7%). Ninety percent of programs had at least one resident on remediation in the last three years. The prevalence of remediation was 4.4%. Indications for remediation ranged from difficulties with one core competency to all six competencies (mean 1.9). The most common were medical knowledge (MK) (63.1% of residents), patient care (46.6%) and professionalism (31.5%). Mean length of remediation was eight months (range 1–36 months). Successful remediation was 59.9% of remediated residents; 31.3% reported ongoing remediation. In 8.7%, remediation was deemed “unsuccessful.” Training year at time of identification for remediation (post-graduate year [PGY] 1), longer time spent in remediation, and concerns with practice-based learning (PBLI) and professionalism were found to have statistically significant association with unsuccessful remediation.

**Conclusion:**

Remediation in EM residencies is common, with the most common areas being MK and patient care. The majority of residents are successfully remediated. PGY level, length of time spent in remediation, and the remediation of the competencies of PBLI and professionalism were associated with unsuccessful remediation.

## INTRODUCTION

Residency training programs have the responsibility to ensure physicians develop the knowledge, skills, and attitudes required to practice medicine independently and to measure trainees’ competency.^1^ It is expected that individual trainees will attain Accreditation Council for Graduate Medical Education (ACGME) Milestones at different stages during their training.^2^ However, some residents will need remediation with additional resources, time and effort not typical of the majority of trainees in order to meet the established standards of each specialty training program. Much work has been done to improve the understanding and assessment of the competencies; however, few studies have addressed the impact of the competencies on remediation or the process of correcting deficiencies in trainees with the goal of graduating competent attending physicians.^3^

When program directors (PDs) identify a resident who requires additional resources to achieve the minimal competency standards in one of the six ACGME domains, it is recommended that they place that resident on a remediation pathway.^4^,^5^ These remediation plans are tailored to the specific deficiencies of each resident, with the goal that the resident will demonstrate competency in those domains prior to graduation. However, a recent study from the members of the Council of Residency Directors (CORD)–Emergency Medicine (EM) Remediation Task Force reported great variation in the definition and management of remediation among EM programs.^4^ The national prevalence of remediation, domains of concern and success rates of remediation in EM are not known.

The primary objective of this study was to determine the prevalence of remediation in EM residencies. Secondary objectives included determining the indications, length, and success rates of remediation across the EM residency programs in the United States. A better understanding of remediation will help programs to recognize possible vulnerable times in residency training, or specific domains of EM practice associated with a higher likelihood of unsuccessful resident remediation.

## METHODS

The study developed an anonymous electronic survey using Surveymonkey™ that was distributed via email directly to all 160 allopathic EM PDs in the spring of 2014 (Appendix 1). We excluded two programs that indicated they were new and did not yet have any residents. Three reminder e-mails were sent to non-responders. The institutional review board reviewed this study and determined it to be exempt.

### Survey Development

To provide content validity evidence, four PDs with more than 25 combined years of experience collaborated to construct the survey. The authors are integrally involved in, and provide content expertise in, the area of remediation practices, given their roles on the CORD Remediation Task Force and long-term experience as PDs and medical education leaders. Further, we formulated survey questions through a joint effort with members of the Remediation Task Force. For response process validity, questions were field tested with educational leadership faculty at the authors’ programs, feedback was gathered, and questions were revised as needed.

The instrument, with specific instructions to include the last three years of data, collected the following information: program demographics; number of residents; number of residents placed on remediation (formal or informal) in the last three years; the post-graduate year (PGY) level of the resident(s) placed on remediation, length of remediation, whether or not the remediation was successful; and the core competency for which the resident was remediated.

The primary objective of this study was to determine the prevalence of remediation in EM residencies. In addition, we looked at the outcome measure of successful remediation of individual residents. Independent variables included program type (PGY-3 vs. PGY-4), training year the resident was placed on remediation, individual core competencies cited as deficient, length of time spent on remediation, and a stratified number of deficient competencies identified. The training year identified combined PGY-3 and PGY-4 into a single “senior resident” category, due to small numbers.

### Outcomes and Data Analysis

Descriptive data were reported. Survey results included program size and total number of residents, which we calculated based on average class size over a three year period in order to obtain the number of residents who were at risk of remediation in the sample. Residents included in the analysis were all individuals with reported outcome data. The results were explored on the basis of inciting factors to place a resident on remediation and also factors associated with successful and unsuccessful remediation. We performed statistical analysis using STATA 12. A multinomial logistic regression model was estimated and presented in Table 1. Covariates included program length, training year resident identified, length of time on remediation, and each of the individual core competencies as identified issues, and grouping of number of identified concerns. We performed model characteristics of area under the ROC curve and Hosmer-Lemeshow test.

## RESULTS

We obtained responses from 126 programs (79.7%). The majority (71%) were three-year programs, while 29% were four-year programs. Six programs were in existence for less than three years. The number of residents per program ranged from six to 84 with a total of 4,711 over the three-year period.

### Remediation Prevalence and Practice

There were a total of 351 residents on remediation in the last three years. Most programs (90%, 113) had at least one resident on remediation during the past three years, while 66% had more than one resident on remediation. The calculated prevalence of remediation in all programs was 4.4%. Remediation periods ranged from one month, while others were greater than three years (in four-year programs). The mean length of successful remediation was 8.0 (SD 5.1) months; for unsuccessful remediation it was 9.9 (SD 8.3) months, and for residents still in progress it was 8.5 (SD 5.3) months. The overall mean of time on remediation in the data was 8.2 (SD 5.5) months.

### Domain of Difficulty and Year of Identification

We found that almost half of residents were identified for remediation (47.9%) during the PGY-2 year, while 26.2% were identified during the PGY-1 year. Respective characteristics of residents placed on remediation by individual competency, training year identification, and number of core competencies cited are provided in Table 2. Of the residents remediated, the three most commonly cited competencies as a concern were patient care (n=155 out of 333; 46.6%), medical knowledge (MK) (n=210 out of 333; 63.1%), and professionalism (n=105 out of 333; 31.5%). Less common competencies reported were communication skills (n=84 out of 333; 25.2%), PBLI (n=40 out of 333; 12.0%), and system-based practice (n=34 out of 333; 10.2%). One to two deficient competencies were most common (72.9%) for residents in remediation (Table 2).

PDs were asked to give specific reasons why residents were placed into remediation/probation status. Here, many individualized specific reasons were cited for changing a resident’s status. However, two comments seemed to recur: performing poorly on the in-training exam (ITE), and “personality flaws,” although many PDs did also comment on the fact that most of those types of issues are not really changeable.

### Successful and Failed Remediation

Successful remediation was common (59.9%) and failure uncommon (8.7%), with many residents’ remediation still in progress (31.3%) and thus the outcome is unknown. The multinomial logistic regression using successful remediation, failure of remediation, and ongoing remediation as the outcomes, and independent variables of program length, training year identified, length of time on remediation, patient care, MK, communication skills, PBLI, system-based practice, professionalism, and number of competencies, resulted in a statistically significant model (p<0.005).

PBLI and professionalism problems were correlated with a decreased likelihood of successful remediation. The training year at time of identification for remediation was found to be statistically significant, with later identification in residency associated with an increased relative chance for success (Table 1). This effect was most clearly demonstrated in PGY-2 vs PGY-1, with residents identified in PGY-1 having a decreased likelihood of successful remediation. Increased length of time spent in remediation was also associated with a decreased likelihood of successful remediation. There was an inverse correlation between year identified and number of competencies identified, meaning PGY-1 had fewer concerning competency domains but it had a more powerful correlation with the outcome of unsuccessful remediation compared to number of competency domains. This resulted in year of identification being significant but not number of competencies. The in-progress outcome was omitted for clarity from Table 1 as it provided no additional statistically significant findings.

## DISCUSSION

Our study found that it is common for EM residencies to place residents on remediation, with 90% of programs reporting at least one resident on remediation in the last three years. More impressively, the data show approximately 4.4% of all EM residents on remediation during the three-year time period with 8% of these residents eventually failing the remediation process. Controlling for other variables, the year of starting remediation (intern year), increased length of remediation, and remediation in the domains of PBLI and professionalism were statistically more likely to have an unsuccessful remediation.

It is common for trainees to be on remediation for deficits in more than one competency domain. This is similar to other studies of internal medicine and pediatric residents.^6^ When looking at the reasons residents were placed into remediation status, grouped by the core competencies, MK was found to be the most common domain for remediation.^5^,^7^

This is likely multifactorial. It may be the easiest core competency deficiency to identify, since almost all EM programs use the ITE.^8^ Further standardized testing can be used to target remediation on MK by implementing an individualized education plan for low scoring residents to improve scores.^9^,^10^ Several studies have found this to be effective.^9^,^10^ While MK may be the most common domain, it was also found to be the most successful core competency to remediate. This high success rate is probably due to the large number of tools available to aid in the remediation process for knowledge gaps. Question banks and board review courses specifically target these issues, so personal remediation plans do not have to be created other than identifying the issue and granting access to such tools. While these are approaches to remediation, Hauer and colleagues called for more research to develop evidence-based strategies for remediation.^11^ System- based practice, PBLI, and professionalism were found to be the least common reasons for residents to be placed on remediation. It is possible that this is due to difficulty with measurement. In particular, professionalism may be reported by private communication rather than an official format such as a rotation evaluation.^12^,^13^ On the other hand, PBLI and professionalism were the competencies least likely to be successful in remediation. However, most of the residents with these deficiencies had problems with other domains as well.

The PGY-2 year was the most common time for residents to be placed on remediation. The etiology of this may again be multifactorial. It is possible that PGY-1s were less likely to be placed on remediation because PDs understand that these residents have not yet developed many skills in the core competencies. Therefore, if problems manifest in the intern year, they were significant. Additionally, many of the intern months are spent in other departments and the ITE results return late in the year. The assessment data may therefore be suboptimal. Further, second-year residents begin to have significant responsibility within the ED, allowing deficits to manifest. However, interns placed on remediation were more likely to fail remediation compared to other years, with up to 20% of interns on remediation being reported as “unsuccessful” remediation.

Residents were found to be on remediation status for a variable length of time. Successful remediation requires time to develop and implement plans, monitor resident progress and allow the resident to demonstrate improvement. Not surprisingly, residents with longer remediation were more likely to be unsuccessful. In addition, it should also be pointed out that when residents are found to be deficient in more than one core competency, their remediation plan should also be multifaceted and should target each deficiency with a specific plan to correct each gap.

While our study found remediation to be common, our results may underestimate the frequency of resident problems. Yao reported that 20% of surveyed PDs of internal medicine residency programs reported fear of litigation and retribution as a reason for avoidance of labeling problem residents as “on remediation.”^14^ In addition, there is a large amount of overlap in the reasons for residents being placed into remediation. It is possible that an individual with a single deficiency in one core competency may be overlooked if the resident is strong in other competencies. Sullivan et al. give the example of the resident who is repeatedly late for conference whose unprofessional behavior may be overlooked if they excel in MK.^12^ These may underestimate the frequency of behaviors that might be considered for remediation and be considered a limitation for this study. Nonetheless, once identified, residents may have multiple areas of concern they need to work on correcting.

Future directions might prospectively identify a cohort of residents on remediation and examine the overlap of domains, determine methods of successful remediation and risk factors associated with failure to remediate.

## LIMITATIONS

This study had several limitations. First, it was a survey-based study with inherent limitations related to interpretation of the questions. We attempted to address the validity issues by building content and response process validity through development and piloting. Since the definitions of remediation and successful remediation are not precisely defined, there may be some variability in responses to these questions. Secondly, the total number of residents possible in the three-year period was calculated based on program size reports. This does not fully account for residents entering or leaving a program during the period and the fact that some residents will not have completed the program, but it still provides a good approximation of remediation frequency.

We compared remediation for different core competencies; however, the majority of remediation plans were for more than one competency. This makes it difficult to draw definitive conclusions about individual core competencies. Additionally, a number of the residents were currently in remediation, and the outcome for these residents is unknown. Finally, it may be difficult to remember exactly which year and what the issues were with the various residents on remediation. It was for this reason that we chose a three-year time frame, but there may be inaccuracy in response.

## CONCLUSION

Resident remediation during EM residency training is common, with close to 90% of programs having at least one resident on remediation in a recent three-year period. The most common areas to remediate are MK and patient care. There is a wide range in length and success of remediation.

## Supplementary Information



## Figures and Tables

**Table 1:** Successful remediation compared to failed remediation. Base category is failure. Area under the Receiver Operating Characteristic (ROC) curve for success vs failure is 0.82, indicating good discriminatory power in the model. Area under the ROC curve for success vs. in progress is 0.44. The Hosmer-Lemeshow test for goodness of fit had a p<0.62, indicating non-statistically significant differences between deciles and therefore an adequate fit to the data.

Success versus failure	Relative risk ratio (standard errors)	95% CI
PGY 3 vs PGY 4 programs	1.16 (0.71)	0.35–3.83
Year identified for remediation		
PGY 2 vs PGY 1	5.15 (3.07)**	1.60–16.56
PGY 3 & 4 vs PGY1	3.29 (2.16)	0.91–11.92
Length of time in remediation in months	0.91 (0.03)*	0.85–0.98
Competency domain		
Patient care	0.04 (0.07)	0.00–1.06
Medical knowledge	0.14 (0.23)	0.01–3.41
Communication skills	0.21 (0.33)	0.01–4.51
Practice based learning	0.03 (0.06)*	0.00–0.96
System based practice	0.20 (0.29)	0.01–3.37
Professionalism	0.03 (0.05)*	0.00–0.66
Number of identified concerns		
Two vs. one competency	25.87 (43.94)	0.93–721.97
Three vs. one competency	115.4 (357.5)	0.27–50,043.33
Four or more vs. one competency	837.7 (4,261)	0.04–17,900,000

*PGY*, post-graduate year

* p<0.05.

** p<0.01.

**Table 2:** Remediation characteristics. Total residents includes all residents with reported outcome data taking into account missing data.

Core competencies*	Number of residents on remediation with this issue	Successful remediation (%)	Unsuccessful remediation (%)	Still in progress (%)
Patient care	155	82 (53.3%)	18 (11.7%)	54 (35.1%)
Medical knowledge	210	127 (61.4%)	16 (7.7%)	64 (30.9%)
Communication skills	84	43 (51.2%)	8 (9.5%)	33 (39.3%)
Practice based learning	40	14 (35.0%)	8 (20.0%)	18 (45.0%)
System based practice	34	16 (47.1%)	6 (17.7%)	12 (35.3%)
Professionalism	105	51 (49.0%)	13 (12.5%)	40 (38.5%)
Issue in 1 competency	149 (45.4%)	102 (67.5%)	11 (7.3%)	36 (23.8%)
Issue in 2 competencies	105 (32.0%)	64 (61.0%)	6 (5.7%)	35 (33.3%)
Issue in 3 competencies	44 (13.3%)	20 (44.4%)	3 (6.7 %)	21 (46.7%)
Issue in 4 or more competencies	30 (9.1%)	10 (33.3%)	8 (26.7%)	12 (40.0%)
PGY 1 remediation outcome	88 (26.8%)	40 (45.5%)	11 (12.5%)	37 (42.0%)
PGY 2 remediation outcome	160 (48.8%)	103 (63.9%)	11 (6.8%)	46 (28.6%)
PGY 3 & 4	80 (24.4%)	54 (67.5%)	5 (6.3%)	21 (26.3%)

*PGY*, post-graduate year

* Number >100% as some residents have more than one competency identified.
