# Supplementary material for: What is the Prevalence and Success of Remediation of Emergency Medicine Residents?
Source: West J Emerg Med. 2015 Oct 22;16(6):839–44. doi: 10.5811/westjem.2015.9.27357 (PMC4651579; doi:10.5811/westjem.2015.9.27357)
Supplement: Supplementary file 1 [file wjem-16-839-s001.pdf]

Anonymous self-report survey
Staten Island University Hospital
Consent for Participation in a Research Study

Study title: WHAT IS THE STATE OF REMEDIATION IN EMERGENCY MEDICINE RESIDENCY PROGRAMS?

This research survey is being conducted by Dr. Weizberg from Staten Island University Hospital on behalf of the CORD Remediation Task Force. We are conducting a research study about the state of remediation in Emergency Medicine residency programs. The following survey should take about 5 minutes to complete.

Participation is voluntary. If you would prefer not to participate, simply submit a blank survey. If you agree to participate, please complete the attached survey. Your responses are anonymous; do not put your name or other identifying information on this survey. We ask that you try to answer all questions. However, if there are any questions that you would prefer to skip, simply leave the answer blank.

Researcher contact information:
Name: Dr. Moshe Weizberg
Title: Residency Director
Dept: Emergency Medicine
Staten Island University Hospital
Phone: 718-226-1548
Email: mweizberg@siuh.edu

This research has been reviewed by the Institutional Review Board (IRB). If you have any questions about your rights as a participant, or if you feel that your rights have been violated, please contact the IRB at 516-321-2100.

1. What is the length of your program?

- ○ 3 years
- ○ 4 years

2. How many total residents are in your program?

[ ]

FOR THE FOLLOWING QUESTIONS, PLEASE INCLUDE BOTH FORMAL AND INFORMAL REMEDIATION

3. In the last 3 years, how many residents in your program have been on remediation?

[ ]

4. For each resident placed on remediation, please indicate the following:

	What PGY year was the resident placed on remediation?	How long was the resident on remediation?	Was the remediation successful?
Resident #1			
Resident #2			
Resident #3			
Resident #4			
Resident #5			
Resident #6			
Resident #7			
Resident #8			
Resident #9			
Resident #10			

5. For each resident placed on remediation, please indicate which core competency they were remediated for? (Check all that apply)

	Patient Care	Medical Knowledge	Interpersonal and Communication Skills	Practice-Based Learning	Systems-Based Practice	Professionalism
Resident #1	☐	☐	☐	☐	☐	☐
Resident #2	☐	☐	☐	☐	☐	☐
Resident #3	☐	☐	☐	☐	☐	☐
Resident #4	☐	☐	☐	☐	☐	☐
Resident #5	☐	☐	☐	☐	☐	☐
Resident #6	☐	☐	☐	☐	☐	☐
Resident #7	☐	☐	☐	☐	☐	☐
Resident #8	☐	☐	☐	☐	☐	☐
Resident #9	☐	☐	☐	☐	☐	☐
Resident #10	☐	☐	☐	☐	☐	☐

6. For each resident, please describe the issues that resulted in their being placed on remediation?

Resident #1

Resident #2

Resident #3

Resident #4

Resident #5

Resident #6

Resident #7

Resident #8

Resident #9

Resident #10

7. Is your program less than 3 years old (since inception)?

- Yes
- No

8. How many years old is your program (since inception)?

9. Please enter any comments below.
